# A rare olive compound oleacein functions as a TrkB agonist and mitigates neuroinflammation both in vitro and in vivo

**DOI:** 10.1186/s12964-024-01691-x

**Published:** 2024-06-04

**Authors:** Daiki Wakasugi, Shinji Kondo, Farhana Ferdousi, Seiya Mizuno, Akira Yada, Kenichi Tominaga, Satoru Takahashi, Hiroko Isoda

**Affiliations:** 1 Tsukuba Life Science Innovation Program (T-LSI), University of Tsukuba, Tsukuba, Ibaraki 305-8572 Japan; 2 Alliance for Research on the Mediterranean and North Africa (ARENA), University of Tsukuba, Tsukuba, Ibaraki 305-8572 Japan; 3 Institute of Life and Environmental Sciences, University of Tsukuba, Tsukuba, Ibaraki 305-8575 Japan; 4 Laboratory Animal Resource Center (LARC) in Transborder Medical Research Center (TMRC), Institute of Medicine, University of Tsukuba, Tsukuba, Ibaraki 305-8575 Japan; 5 Open Innovation Laboratory for Food and Medicinal Resource Engineering (FoodMed-OIL), National Institute of Advanced Industrial Science and Technology (AIST), Tsukuba, Ibaraki 305-0821 Japan; 6 Interdisciplinary Research Center for Catalytic Chemistry, National Institute of Advanced Industrial Science and Technology (AIST), Tsukuba Central 5, 1-1-1 Higashi, Ibaraki, 305-8565 Japan

**Keywords:** Oleacein, Neuroinflammation, BDNF, TrkB, Whole-transcriptomics, SH-SY5Y cells, LPS, Surface plasmon resonance (SPR) assay, Bioluminescence imaging, Bdnf-IRES-AkaLuc mice, Depression

## Abstract

**Background:**

Neuroinflammation is widely acknowledged as a characteristic feature of almost all neurological disorders and specifically in depression- and anxiety-like disorders. In recent years, there has been significant attention on natural compounds with potent anti-inflammatory effects due to their potential in mitigating neuroinflammation and neuroplasticity.

**Methods:**

In the present study, we aimed to evaluate the neuroprotective effects of oleacein (OC), a rare secoiridoid derivative found in extra virgin olive oil. Our goal was to explore the BDNF/TrkB neurotrophic activity of OC and subsequently assess its potential for modulating neuroinflammatory response using human neuroblastoma cells (SH-SY5Y cells) and an in vivo model of depression induced by lipopolysaccharide (LPS)-mediated inflammation.

**Results:**

In SH-SY5Y cells, OC exhibited a significant dose-dependent increase in *BDNF* expression. This enhancement was absent when cells were co-treated with inhibitors of BDNF's receptor TrkB, as well as downstream molecules PI3K and MEK. Whole-transcriptomics analysis revealed that OC upregulated cell cycle-related genes under normal conditions, while downregulating inflammation-associated genes in LPS-induced conditions. Furthermore, surface plasmon resonance (SPR) assays demonstrated that OC exhibited a stronger and more stable binding affinity to TrkB compared to the positive control, 7,8-dihydroxyflavone.

Importantly, bioluminescence imaging revealed that a single oral dose of OC significantly increased BDNF expression in the brains of Bdnf-IRES-AkaLuc mice. Furthermore, oral administration of OC at a dosage of 10 mg/kg body weight for 10 days significantly reduced immobility time in the tail suspension test compared to the LPS-treated group. RT-qPCR analysis revealed that OC significantly decreased the expression of pro-inflammatory cytokines *Tnfα*, *Il6*, and *Il1β*, while simultaneously enhancing *Bdnf* expression, as well as both pro and mature BDNF protein levels in mice hippocampus. These changes were comparable to those induced by the positive control antidepressant drug fluoxetine. Additionally, microarray analysis of mouse brains confirmed that OC could counteract LPS-induced inflammatory biological events.

**Conclusion:**

Altogether, our study represents the first report on the potential antineuroinflammatory and antidepressant properties of OC via modulation of BDNF/TrkB neurotrophic activity. This finding underscores the potential of OC as a natural therapeutic agent for depression- and anxiety-related disorders.

## Introduction

Depression is a serious mental health disorder affecting approximately 3.8% of the global population, leading to more than 700,000 deaths due to suicide every year [1]. Although there are numerous antidepressants available, their effectiveness is often limited by severe side effects, the lengthy duration required for therapeutic effects, treatment resistance, and drug-drug interactions in patients with comorbid conditions [2, 3]. Given these challenges, there is an urgent need to explore new, more effective treatments for depression and mental health, incorporating different mechanisms of action.

In recent years, there has been renewed interest in the intricate relationship between neuroinflammation and the neurogenic hypotheses of depression. These hypotheses suggest that the hippocampus, particularly in the form of adult hippocampal neurogenesis, plays a significant role in the pathophysiology of depression. Animal studies have demonstrated that chronic stress diminishes adult hippocampal neurogenesis [4, 5]. On the other hand, increasing adult neurogenesis alone is adequate to alleviate anxiety and depression-related behaviors in mice [6, 7]. In this context, brain-derived neurotrophic factor (BDNF) emerges as a key molecule with the ability to regulate the molecular interactions between neuroinflammation and neurogenesis in the hippocampus, presenting a potential avenue for addressing both phenomena [8]. BDNF is a neuropeptide that acts through its high-affinity receptor, tropomyosin receptor kinase-B (TrkB). It plays a critical role in maintaining neuroplasticity and regulating neuronal development, differentiation, and survival. Pro-inflammatory cytokines or lipopolysaccharide (LPS) has been demonstrated to significantly reduce mature BDNF levels in brain regions, including hippocampus [9]. Recent research has further linked BDNF/TrkB signaling to depression. It has been reported that a TrkB agonist, 7,8-dihydroxyflavone (7,8-DHF), a naturally occurring flavone, exhibited antidepressant effects in LPS-induced depression-like phenotypes, whereas a TrkB antagonist, ANA-12, blocked this effect [10, 11]. These findings collectively suggest that targeting the BDNF-TrkB signaling pathway using natural bioactive compounds could offer a novel and safe therapeutic approach for depression.

In this context, olive phenolic compounds, such as oleuropein (OP) [12, 13] and hydroxytyrosol (HT) [14, 15], have been reported to exert neuroprotective effects by enhancing BDNF expression and reducing the expression of proinflammatory cytokines in the hippocampus of various stress-induced rodent models. Another olive compound, oleocanthal, has been reported to possess potent anti-inflammatory properties similar to ibuprofen [16]. This is particularly interesting, as systemic administration of ibuprofen has been reported to exert anxiolytic-like activity in animal models by inhibiting proinflammatory cytokine expression and restoring BDNF expression [17]. In this regard, a rare olive compound, oleacein (OC), which shares a similar dialdehyde structure with oleocanthal but differs by an additional phenolic hydroxyl group, has recently gained attention for its potent bioactivities. However, due to its relatively low occurrence, its biological functions have not been as extensively investigated as those of oleocanthal or other olive compounds. Nevertheless, there has been a resurgence of interest in OC following the discovery of a one-step synthesis method from OP [18].

In the present study, we aimed to comprehensively investigate the antidepressant-like activity of OC and its potential to alleviate neuroinflammation by modulating BDNF/TrkB signaling, utilizing a series of in vitro and in vivo experiments.

## Methods

### SH-SY5Y cell culture

The human neuroblastoma clone SH-SY5Y cell line was obtained from the American Type Culture Collection (ATCC®, Manassas, VA, USA). The cells were cultured in a 1:1 (v/v) mixture of Dulbecco's modified Eagle's medium and Ham's F-12 medium (Gibco, Grand Island, NY, USA) supplemented with 15% fetal bovine serum (FBS) (Sigma-Aldrich, St. Louis, MO, USA), MEM nonessential amino acids (FUJIFILM Wako Pure Chemical Corporation, Osaka, Japan), and 1% penicillin (5000 μg/mL)-streptomycin (5000 IU/mL) solution (Sigma-Aldrich) as a growth medium in 75 cm^2^ flask. The flasks were maintained under an atmosphere of 5 % CO2/95 % humidified air at 37°C. The medium was changed every two days. The proliferated SH-SY5Y cells were utilized either for subsequent gene expression analysis or cryopreserved in liquid nitrogen as stocked cells.

### Treatments of oleacein to SH-SY5Y cells and RNA isolation

OC was synthesized following the methods previously reported [18] and provided for this study. For gene expression analysis, SH-SY5Y cells (2 × 10^6^ cells/5 ml growth medium) were seeded into 60 mm dishes. OC was dissolved in dimethyl sulfoxide (DMSO, FUJIFILM Wako Pure Chemical Corporation, Osaka, Japan) and further diluted in serum-free Eagle's minimum essential medium (OPTI-MEM, Gibco) to achieve treatment concentrations ranging from 1 to 50 µM. Similarly, OPTI-MEM containing 0 µM OC and 0.05% DMSO was prepared as the control. Twenty-four hours post-seeding, the SH-SY5Y cells were cultured in OPTI-MEM for an additional 24 hours. Subsequently, they were subjected to a 24-hour incubation period either without OC or with varying concentrations of OC for subsequent gene expression analysis.

Additionally, inhibitor experiments were conducted to analyze the target of OC in the TrkB/BDNF pathways. After seeding SH-SY5Y cells in 60 mm dishes, they were shifted from growth medium to OPTI-MEM and allowed to incubate for 24 hours. Subsequently, the cells were treated with 10 µM OC and concurrently co-treated with the same concentration of one of the inhibitors: 10 µM ANA-12 (from Selleck Biotech, Kanagawa, Japan) acting as a TrkB antagonist, 10 µM LY294002 (from ChemScene, NJ, USA) acting as a phosphatidylinositol 3-kinase (PI3K) inhibitor, or 10 µM PD98059 (from ChemScene) acting as a mitogen-activated protein kinase (MEK) inhibitor for an additional 24 hours.

To assess the responsiveness of OC-treated SH-SY5Y cells to inflammation, lipopolysaccharide (LPS) was introduced to the OC-treated cells. Following seeding of SH-SY5Y cells in 60 mm dishes, the cells were transitioned from growth medium to OPTI-MEM and incubated for 24 hours. Subsequently, 10 µM OC and 5 µg/ml LPS (FUJIFILM Wako Pure Chemical Corporation) were co-administered to the cells for an additional 24 hours in OPTI-MEM.

For gene expression analysis (real-time PCR and DNA microarray), total RNA was isolated from the treated SH-SY5Y cells using ISOGEN (Nippon Gene, Tokyo, Japan) according to the manufacturer's instructions. The quantity and quality of RNA were assessed using a NanoDrop 2000 spectrophotometer (Thermo Fisher Scientific, Waltham, MA, USA).

### Quantitative real-time PCR

Real-time PCR was conducted to analyze the gene expression in SH-SY5Y cells and the brain hippocampus of mice. The TaqMan probe (Thermo Fisher Scientific) was utilized for the quantification of gene expression. A cDNA solution was synthesized using a superscript IV VILO master mix (Thermo Fisher Scientific) following the manufacturer’s instructions. For the quantification of transcript amounts, TaqMan real-time RT-PCR amplification reactions were carried out using the Applied Biosystems 7500 Fast Real-Time System (Thermo Fisher Scientific). All primer sets and the TaqMan Universal PCR Master Mix were procured from Thermo Fisher Scientific, including GAPDH (Hs02786624 and Mm02619580_g1) as an internal control, and the following targets: BDNF (Hs03298540_m1 and Mm01329577_g1), *Tnf* (Mm00443258_m1), *Il6* (Mm00446190_m1), and *Il1β* (Mm00434228_m1).

### Bdnf-IRES-AkaLuc-mice

We selected a sequence (5´-ATG AAG TTT ATA CAG TAC AG-3´) very close downstream of the termination codon of the *Bdnf* as the CRISPR target. We purchased synthetic crRNA containing this target in sequence from IDT (Iowa, US). In the pBdnf-IRES-Akaluc, we placed the IRES-Akaluc sequence between the 5´ and 3´ homology arms. The 5´-homology arm is the genomic region from 831 bp upstream of the *Bdnf* termination codon to 128 bp downstream of the *Bdnf* termination codon, and the 3´-homology arm is the genomic region from 129 to 1,244 bp downstream of the Bdnf termination codon.

The CRISPR-Cas9 ribonucleoprotein complex and each donor DNA were microinjected into zygotes of C57BL/6J mice (Jackson Laboratory Japan, Kanagawa, Japan) according to our previous report. Subsequently, microinjected zygotes were transferred into oviducts in pseudopregnant ICR female (Jackson Laboratory Japan, Kanagawa, Japan) and newborns were obtained.

### AkaLumine-AkaLuc bioluminescence imaging in vivo

To observe noninvasive BDNF expression in vivo, AkaLumine-AkaLuc bioluminescence imaging (AkaBLI) was conducted using Bdnf-IRES-AkaLuc mice. AkaLumine-HCl (TokeOni) was procured from Fujifilm Wako. AkaLumine-HCl was dissolved in saline and administered intraperitoneally at a dosage of 75 µmol/kg body weight. The AkaLumine solution was prepared on ice. Following administration, images were captured using the In Vivo Imaging system (IVIS) at a rate of one image per minute for 20 minutes, and luminescence intensity was monitored over time. The peak luminescence intensity observed over the 20-minute period was recorded as the value for each individual mouse.

### SPR-based intermolecular binding assay

To prepare the TrkB-immobilized sensor chip, the extracellular domain (ECD) of human recombinant TrkB protein (10047-H02H, Sino Biological, Inc. Beijing, China) was reconstituted in distilled water to a concentration of 0.25 mg/ml. It was then diluted in 10 mM sodium acetate (pH 4.5) (Cytiva, Tokyo, Japan) to a concentration of 50 μg/ml. The TrkB protein solution was allowed to contact the CM5 sensor chip (Cytiva) for 7 minutes and then immobilized using an amine coupling kit containing 1-ethyl-3-(3-dimethylaminopropyl) carbodiimide hydrochloride (EDC), N-hydroxysuccinimide (NHS), and 1M ethanolamine hydrochloride-NaOH pH 8.5 (Cytiva). The immobilization step ran for 120 seconds. Chip regeneration was carried out using 50 mM NaOH. The immobilized protein reached a response unit (RU) value of 10916.7.

For the preparation of analytes, OC, 7,8-DHF (Tokyo Chemical Industry, Tokyo, Japan), and 5,7-dihydroxyflavone (chrysin) (Cayman Chemical, Ann Arbor, MI, USA) were dissolved in DMSO to a concentration of 100 mM each. 7,8-DHF and chrysin were utilized as positive and negative controls, respectively, for binding affinity to the TrkB protein. These stock solutions were then diluted in HBS-EP+ buffer (containing 10 mM HEPES, 0.15 M NaCl, 3 mM EDTA, and 0.05% (v/v) surfactant P20) (Cytiva) to a concentration of 500 µM. Additionally, solutions of the analytes at 500 µM were further diluted in HBS-EP+ buffer with 0.5% (v/v) DMSO to concentrations of 31.25, 62.5, 125, and 250 µM.

The analyte solution (OC, 7,8-DHF, or chrysin) was injected onto the TrkB ECD-immobilized sensor chip set in a Biacore X100 system (Cytiva) at a flow rate of 30 μl/min (contact time: 120 s, dissociation time: 200 s).

Manual run and single-cycle kinetics modes were employed to determine the response unit (RU) values and dissociation rate (Kd) values, respectively. Data analysis was conducted using Biacore X100 evaluation software version 1.0+ (Cytiva).

### Docking simulation

Docking simulation of human TrkB (PDB ID: 1HCF) and OC was performed in Molegro Virtual Docker (MVD) [19]. Following the previous works [20, 21], we decided to dock OC to the region d5 of TrkB with which 7,8-DHF would interact. PDB ID: 1HCF was imported into MVD and all but chain X containing the ligand binding site (d5) was deleted. Chiu et al. [20] showed that, the amino acid residue within 10 Å from the docking result was Lys312, Pro313, Ala314, Leu315, Thr332, Lys333 and Ile334. The central coordinate of these amino acid residues was X: -17.70, Y: 11.36, Z: 16.24. Accordingly, the docking area was set within a radius of 15 Å from the above coordinates. In this docking simulation, MolDock Optimizer as the docking algorithm and MolDock Score as the scoring function were used [19].

### Oral administrations to ICR mice

The animal care and experimental procedures conducted in this study were approved by the University of Tsukuba Ethics and Animal Welfare Committee (22-378). Male 8-week-old ICR mice were procured from Jackson Laboratory Japan (Kanagawa, Japan). The mice were individually housed in cages and maintained under controlled environmental conditions, including a room temperature of 21-23°C, a 12-hour light/dark cycle, humidity ranging from 50-55%, and ad libitum access to food and water. Following a one-week acclimation period to the laboratory conditions, the experimental procedures commenced.

After the acclimation period, the mice were orally administered saline, 10 mg/kg body weight fluoxetine (Flux) (FUJIFILM Wako Pure Chemical Corporation) as a positive control, or 10 mg/kg body weight OC daily for ten consecutive days. On the 10^th^ day of oral administration, half of the saline-treated group received a single intraperitoneal injection of saline (control group, *n* = 8), while the other half received a single intraperitoneal injection of 0.85 mg/kg body weight LPS (LPS group, *n* = 8). Similarly, both the Flux and OC groups received the LPS injection (Flux group and OC group) (*n* = 8 each) on the 10^th^ day following the commencement of oral administration.

### Tail suspension test (TST)

Twenty-four hours following the intraperitoneal injection of LPS into the ICR mice, the tail suspension test (TST) was conducted as described in a previous study [22] with slight modifications. Briefly, each mouse was individually suspended for 6 minutes at a point 2 cm from the tail tip using a clip (Yamashita Giken, Tokushima, Japan) in a white box measuring 30 × 15 × 50 cm (length × width × height). The immobility time, indicative of behavioral despair akin to clinical depression in humans, was recorded and measured during the final 4 minutes of the 6-minute suspension period using SMART 3.0 video tracking software (Panlab, Barcelona, Spain). Mice were considered immobile only when they ceased movement of their limbs, head, and body.

### Isolation of RNA and protein from the *hippocampus* of ICR mice brains

After the completion of the TST, all ICR mice were euthanized via cervical dislocation, and their brains were collected. The brains were rapidly frozen using liquid nitrogen and stored at −80°C until further use. Total RNA for gene expression analysis (real-time PCR and DNA microarray) was extracted from the hippocampus of the mice brains using ISOGEN (Nippon Gene). The quantity and quality of RNA were assessed using a NanoDrop 2000 spectrophotometer (Thermo Fisher Scientific).

For protein expression analysis (ELISA), total protein was extracted from the hippocampus of the mice brains using RIPA buffer (Sigma-Aldrich) supplemented with 1% (v/v) protease inhibitor cocktail (Sigma-Aldrich), following the manufacturer's instructions. The protein concentrations were determined using the Pierce BCA Protein Assay Kit (Thermo Fisher Scientific) and a Varioskan LUX multimode microplate reader (Thermo Fisher Scientific) at a wavelength of 562 nm.

### DNA microarray analysis

DNA microarray analyses were conducted on duplicate RNA samples from SH-SY5Y cells across the following groups: control, OC-treated group (OC), LPS-induced group (LPS), and OC-treated and LPS-induced group (OC+LPS). For the in vivo study, microarray analysis was performed on RNA samples extracted from the mice hippocampus of the following groups: saline-administered control, saline-administered and LPS-injected (LPS), and OC-administered and LPS-injected (OC+LPS) groups. GeneChip® Whole Transcript (WT) PLUS microarray reagents and kits (ThermoFisher Scientific, Waltham, MA, USA) were utilized according to the manufacturer's recommended protocols.

To summarize the procedure, cDNA synthesis was initially carried out using 100 ng of RNA solutions. Subsequently, cRNA was generated through in vitro transcription of the cDNA, followed by purification and reverse transcription steps. Finally, single-stranded cDNA (ss-cDNA) was synthesized, purified, fragmented, and labeled as per the manufacturer's instructions.

The Clariom S array (Human and Mouse arrays for the in vitro and in vivo samples, respectively; ThermoFisher Scientific, Waltham, MA, USA) was employed for the DNA microarray hybridization. The GeneChip™ Fluidics Station (ThermoFisher Scientific) was utilized to hybridize the cartridge array, and the resulting hybridized arrays were scanned using the GeneChip Scanner (ThermoFisher Scientific, Waltham, MA, USA).

### Microarray data processing

After obtaining raw image data from the scanning process, we conducted subsequent analysis using the Transcriptome Analysis Console (TAC) software, version 4.0.2 (ThermoFisher Scientific, Waltham, MA, USA). To ensure robustness, the raw data were normalized using the signal space transformation robust multi-chip analysis (SST–RMA) algorithm. Gene-level analysis was then performed using the Limma Bioconductor package included with TAC 4.0.2.

To identify differentially expressed genes (DEGs), we applied a one-way ANOVA followed by an empirical Bayes correction. To further refine the analysis and eliminate non-significant signals, we set a detected above background (DABG) cutoff of 0.05. Additionally, we conservatively set the positive vs negative area under the curve (AUC) value at greater than 0.7 to focus on genes with strong discriminatory potential.

The ultimate selection of DEGs relied on rigorous filter criteria, necessitating a *P* value below 0.05 (computed using one-way between-subject ANOVA) and a fold change (FC) exceeding 2 (in linear space).

### Microarray data analysis

Volcano plots were generated using the VolcaNoseR web application [23]. Gene ontology (GO) overrepresentation analyses were conducted using the web-based tool Metascape (v3.5.20230101, http://metascape.org) [24]. All input gene lists were consolidated into a single list to construct a comprehensive protein-protein interaction (PPI) network. Subsequently, the MCODE algorithm was applied to identify densely connected protein neighborhoods, with the top enriched networks (MCODE 1) being presented.

Gene set enrichment analysis (GSEA) and generic PPI networks were constructed utilizing the NetworkAnalyst tool (version 4.3.2) [25]. PPI interactions were predicted using the STRING Interactome database [26]. Top genes that co-express with BDNF were identified with the ARCHS4 RNA-seq gene-gene co-expression analysis [27].

The HumanBase public database (URL: https://hb.flatironinstitute.org) and its analytical tool 'Module' were utilized to identify functionally clustered modules by the DEGs based on shared k-nearest-neighbors (SKNN) and the Louvain community-finding algorithm [28]. Genes with a co-membership score > 0.9 were assigned to clusters. The resulting modules underwent functional enrichment analysis using genes annotated to GO biological process (GOBP) terms.

Heatmaps were generated using the online tool Morpheus (https://software.broadinstitute.org/morpheus), while Venn diagrams were created using an online tool (https://bioinformatics.psb.ugent.be/webtools/Venn/).

### ELISA

ProBDNF and mBDNF levels in mouse brain hippocampus were measured using a commercially available ELISA kit (Funakoshi, Japan) according to the manufacturer's instructions. Briefly, ProBDNF and mBDNF levels in the brain were measured using protein samples or standards. After treatment with antibodies, a second incubation with streptavidin-horseradish peroxidase conjugate solution was performed for 60 min. After the addition of substrate and stopping solution, ProBDNF and mBDNF levels were measured by absorbance at 450 nm; ProBDNF and mBDNF levels were normalized to the protein concentration using Pierce BCA Protein Assay (Thermo Fisher Scientific).

### Statistical analysis

Data were analyzed using GraphPad Prism (version 8.0, GraphPad Software Inc., San Diego, CA) and SPSS ver. 26 (Armonk, NY: IBM Corp). Data are expressed as mean ± standard error of the mean (SEM) unless otherwise noted. Shapiro-Wilk test was conducted to determine the normal distribution of the data. For two-group comparisons, significant difference tests were conducted using Student's t-test, while for comparisons among multiple groups, one-way analysis of variance (ANOVA) was employed, followed by Tukey’s post hoc test. Differences were considered statistically significant at a value of *P <* 0.05.

## Results

### Oleacein treatment increased the expression of *BDNF* in the SH-SY5Y cells

**Fig. 1:**
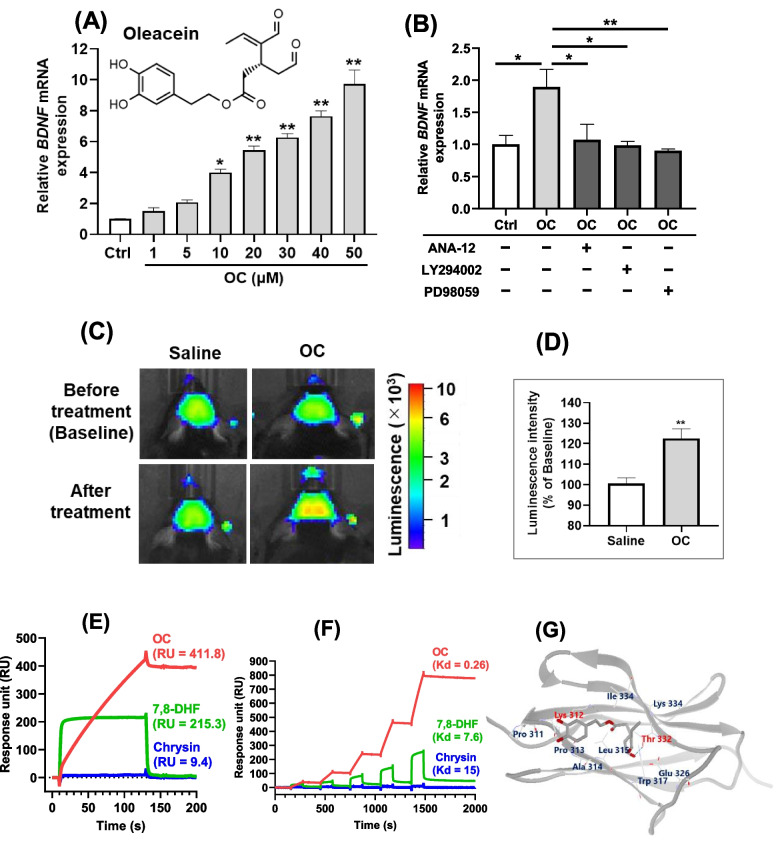
OC increased the BDNF expressions *in vitro and in vivo,* and exhibited the binding affinity to TrkB receptor. **A** *BDNF* mRNA expressions in SH-SY5Y cells treated with OC in the concentration range of 1-50 µM and **B** under co-treatment of 10 µM OC with 10 µM ANA-12 (TrkB selective antagonist), 10 µM LY294002 (PI3K inhibitor), or 10 µM PD98059 (MEK inhibitor) using quantitative real-time PCR. **C** Images of AkaBLI in Bdnf-IRES-AkaLuc mice before (baseline) and 8h after oral administration of OC at a dose of 10 mg/kg bw and **D** their rate of increase in the luminescence intensity after OC treatment compared to the baseline. **E** Binding affinity between 500 µM OC and TrkB receptor and **F** its single-cycle kinetics analysis using OC at the concentrations of 31.25, 62.5, 125, 250, and 500 µM using SPR-based assay of intermolecular interaction (Biacore). The affinities of 7,8-DHF and chrysin were used as positive control and negative control, respectively. Each value represents the mean ± SE for *n* = 4 in each group for SH-SY5Y cells and Bdnf-IRES-AkaLuc mice. Significant difference from the control group at ^*^P < 0.05 and ^**^P < 0.05 by one-way ANOVA followed by Tukey’s post hoc test for SH-SY5Y cells. Significant difference from the saline group at ^**^P < 0.01 by unpaired two-tailed Student’s t-test for Bdnf-IRES-AkaLuc mice. **G** Docking result of Oleacein to the d5 region of TrkB. The top 10 amino acids closest to the position of the docking results are shown. Gray: Protein TrkB (PDB ID: 1HCF). Red: Amino acid residues predicted to form hydrogen bonds. Light blue dotted line: hydrogen bond (Amino acid residues predicted to form hydrogen bonds are also indicated by thin lines). The image was generated with the Molegro Virtual Docker (MVD)

First, we investigated the effect of OC on BDNF mRNA expression in SH-SY5Y cells. Cells were treated with OC for 24 hours, and gene expression analysis of BDNF was conducted using real-time PCR. Treatment with OC resulted in a significant and dose-dependent increase in the expression of the *BDNF* in SH-SY5Y cells, starting from a concentration of 10 µM (Fig. 1A).

BDNF signals through its high-affinity receptor tropomyosin receptor kinase B (TrkB) and influences neuronal growth and differentiation by activating various signaling pathways, notably the mitogen-activated protein kinase (MAPK) and PI3K/Akt pathways. Therefore, we investigated the effects of OC on BDNF expression in the presence of three inhibitors: ANA-12 (a TrkB inhibitor), LY294002 (a PI3K inhibitor), or PD98059 (a MEK inhibitor). We found that subsequent co-administration of OC with these inhibitors resulted in the inhibition of the OC-induced upregulation of BDNF expression in the cells (Fig. 1B).

### A single oral dose of Oleacein increased the protein expression of BDNF in Bdnf-IRES-AkaLuc mouse brain

We utilized bioluminescence imaging (BLI) to non-invasively and longitudinally track and visualize BDNF expression [29] in the living mouse brain before and after OC treatment. Specifically, we employed the Akaluciferase (Akaluc) BLI system known for its robust signal strength [30].

Initially, the mice were orally administered a single oral dose of saline. Eight hours later, TokeOni (AkaLumine-HCl) was intraperitoneally injected at a dose of 75 µmol/kg bw to induce luminescence of the BDNF protein in the mouse brains, and the baseline luminescence intensities were recorded using the IVIS instrument. After one week, the mice were orally administered a single dose of OC at 10 mg/kg bw. Subsequently, TokeOni was again injected intraperitoneally, and the luminescence intensities of the brains were measured. The rate of increase in BDNF expression was calculated based on the luminescence intensity before (baseline) and after OC administration. A single oral administration of OC significantly augmented the expression of BDNF protein in mouse brain compared to the saline group (Fig. 1C, D).

### OC exhibited higher binding affinity to TrkB receptor compared to 7,8-DHF

To detect the binding affinity of OC to TrkB-ECD, the OC, 7,8-DHF (positive control), and chrysin (negative control) were injected and reacted to a sensor chip immobilized with TrkB-ECD in a manual run mode of the Biacore X100. The binding affinity of OC to TrkB-ECD (411.8 response unit; RU) was higher than that of 7.8-DHF (RU = 215.3) and chrysin (RU = 9.4) (Fig. 1E). Additionally, after the injection was stopped, 7,8-DHF (and chrysin) began to dissociate immediately, while that of OC was remarkably slower. To calculate a dissociation rate constant (Kd) of OC, the compounds were injected into the TrkB-ECD-fixed sensor chip in a mode of single-cycle kinetics in the Biacore X100. The Kd value of OC (Kd = 0.26) was lower than those of 7,8-DHF (Kd = 7.8) and Chrysin (Kd = 15.0) (Fig. 1F).

### Molecular interactions of oleacein with TrkB by docking computation

The interactions between OC and the TrkB receptor were analyzed using a molecular docking approach. The structure of TrkB-domain5 (TrkB-D5) was subjected to OC binding in silico to explore the optimal binding conformation of OC. Our study revealed that the binding site of TrkB-D5 comprised Trp317, Ile334, Leu324, Glu326, and Thr332 residues, forming hydrogen bonds (Fig. 1G).

### Transcriptomics analysis revealed oleacein modulated the expression of cell cycle-associated genes under normal condition in SH-SY5Y cells

**Fig. 2:**
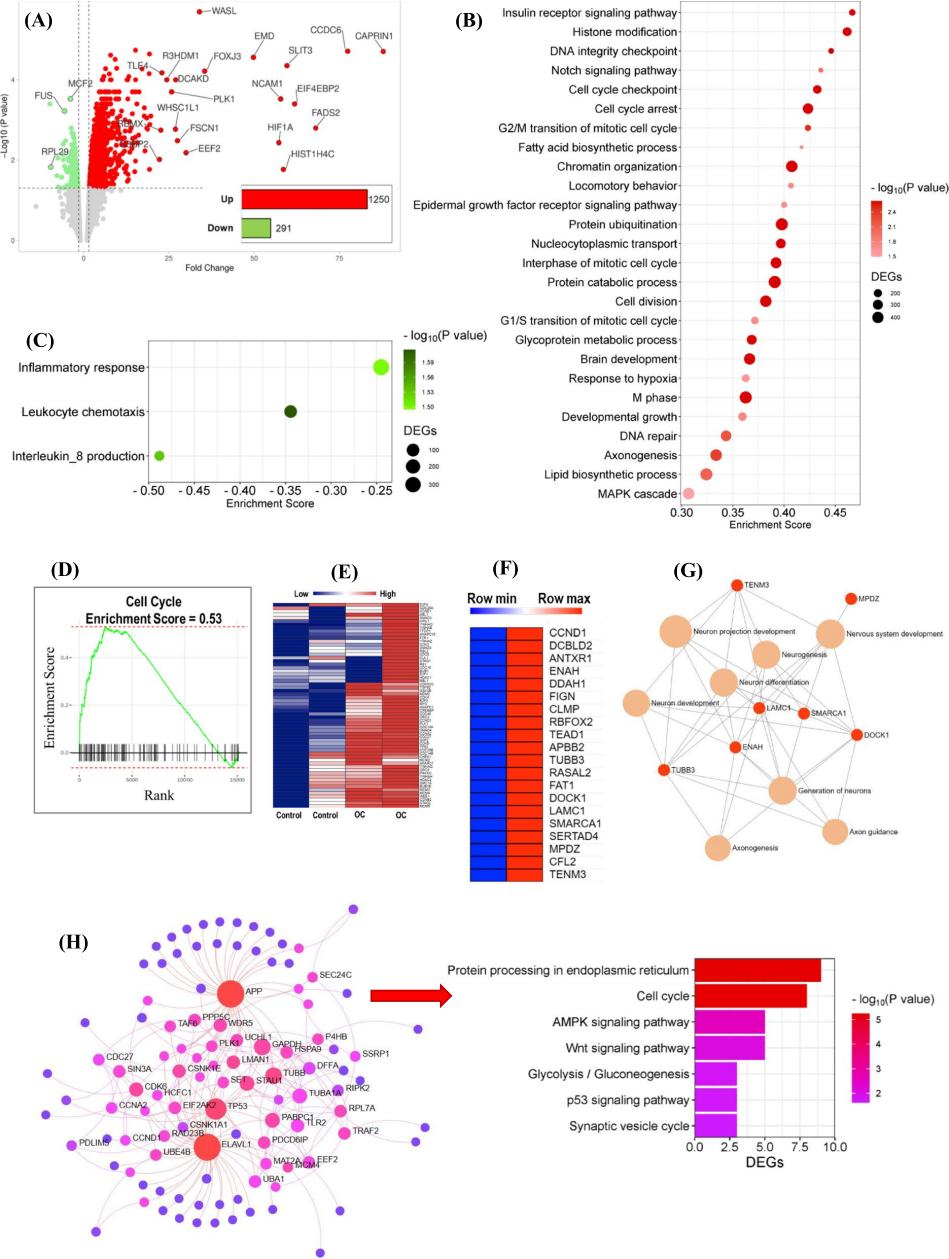
Transcriptomic response of oleacein in SH-SY5Y cells under normal condition. Cells were exposed to 10 µM of oleacein (OC) for 24 hours, followed by RNA isolation from both OC-treated (OC) and untreated (control) conditions, which were subsequently subjected to microarray analysis. **A** A volcano plot illustrating the differentially expressed genes (DEGs) between OC-treated and control SH-SY5Y cells. The vertical axis (y-axis) represents the −log10 *P* value, while the horizontal axis (x-axis) displays the linear fold change. Upregulated genes are depicted in red dots, whereas downregulated genes are shown in green dots. The top 20 DEGs with the largest fold changes are highlighted, along with inset bar graph indicating the number of DEGs. The red bar denotes the count of upregulated DEGs, while the green bar represents the count of downregulated genes. Bubble plots illustrate significantly enriched biological process gene ontology (GOBP) terms, with **B** showing activated GOBP terms and **C** displaying inhibited GOBP terms. The color code represents the significance value (−log10 (*P* value)), the bubble size indicates the number of DEGs in each term, and the x-axis represents the enrichment score. **D** Gene Set Enrichment Analysis (GSEA) plots depict the predicted top activated KEGG pathway, Cell cycle. Heatmaps demonstrate the average signal intensity of **E** 'Cell cycle'-associated genes identified through GSEA analysis, and **F** genes co-expressed with *BDNF*, as identified through ARCHS4 RNA-seq gene-gene co-expression analysis. **G** A bipartite network reveals the interactions between *BDNF*-coexpressed DEGs in OC and the significantly enriched GOBP functional networks associated with them. **H** The PPI network displays the top enriched functional module clustered using the WalkTrap algorithm and the bar graph presents the top enriched GOBPs within the WalkTrap module

In the Clariom S Assay Human Array, a total of 21,452 probe sets were identified. Among them, 1,250 genes were upregulated, while 291 genes were downregulated. The volcano plot in Fig. 2A illustrates the significantly expressed genes between OC-treated and control SH-SY5Y cells, with the top 20 differentially expressed genes (DEGs) featuring prominently. Notably, the top upregulated DEG was cell cycle-associated protein 1 (*CAPRIN1*, FC = 87.87), while the top downregulated DEG was ALF transcription elongation factor 4 (*AFF4*) with FC = −9.93, followed by ribosomal protein L29 (*RPL29*, FC = −9.68).

To predict OC-induced biological events in SH-SY5Y cells, gene set enrichment analysis (GSEA) was conducted, considering the entire distribution of changes in gene expression and ranking the genes by fold change. Significantly enriched gene ontology biological process (GOBP) terms are depicted in the bubble plots in Fig. 2B and C. Top enriched terms were determined based on enrichment score (ES), with positive scores indicating upregulation/activation and negative scores indicating downregulation/inhibition. The upregulated GOBP terms included insulin receptor signaling pathway, Notch signaling pathway, fatty acid biosynthetic process, locomotory behavior, epidermal growth factor receptor (EGFR) signaling pathway, and brain development. Importantly, several cell cycle-related GOBP terms were predominant in the upregulated GOBP term lists, such as cell cycle checkpoint, positive regulation of cell cycle, cell cycle arrest, interphase of mitotic cell cycle, M phase of mitotic cell cycle, G1/S transition of mitotic cell cycle, and cell division. KEGG pathway enrichment analysis showed that 'cell cycle' was significantly upregulated with ES = 0.4673 and *P* value = 0.00005 (Fig. 2D). Indeed, several cell cycle-associated genes exhibited significantly higher expression in OC-treated SH-SY5Y cells compared to non-treated control cells, as shown in the heatmap in Fig. 2E. Conversely, inflammatory response, leucocyte chemotaxis, and interleukin 8 (IL8) production were downregulated (Fig. 2C). Further details of the GSEA analysis can be found in Additional file 1.

Next, the effects of OC on BDNF coexpressed genes were examined. The top 200 genes co-expressed with BDNF were identified using ARCHS4 RNA-seq gene-gene co-expression matrix, and their expression pattern in OC-treated cells was compared with that of control group cells. A total of 19 BDNF coexpressed genes were found to be significantly upregulated (*P <* 0.05, FC > 2) by OC treatment (Fig. 2F). Furthermore, bipartite functional network analysis revealed that BDNF-coexpressed DEGs in the OC-treated group were mainly closely interacting gene sets significantly associated with axon guidance, neuron differentiation, neuron projection development, axonogenesis, and neurogenesis (Fig. 2G).

Subsequently, the WalkTrap community detection algorithm was applied to the generic protein-protein interaction (PPI) network to identify the top functional module enriched by OC treatment (Fig. 2H). GOBP functional network enrichment analysis highlighted cell cycle as the most consistent term, followed by the AMPK signaling pathway, Wnt signaling pathway, glycolysis, and synaptic vesicle cycle.

### DNA microarray analysis showed oleacein mitigated LPS-induced inflammatory response in SH-SY5Y cells

**Fig. 3:**
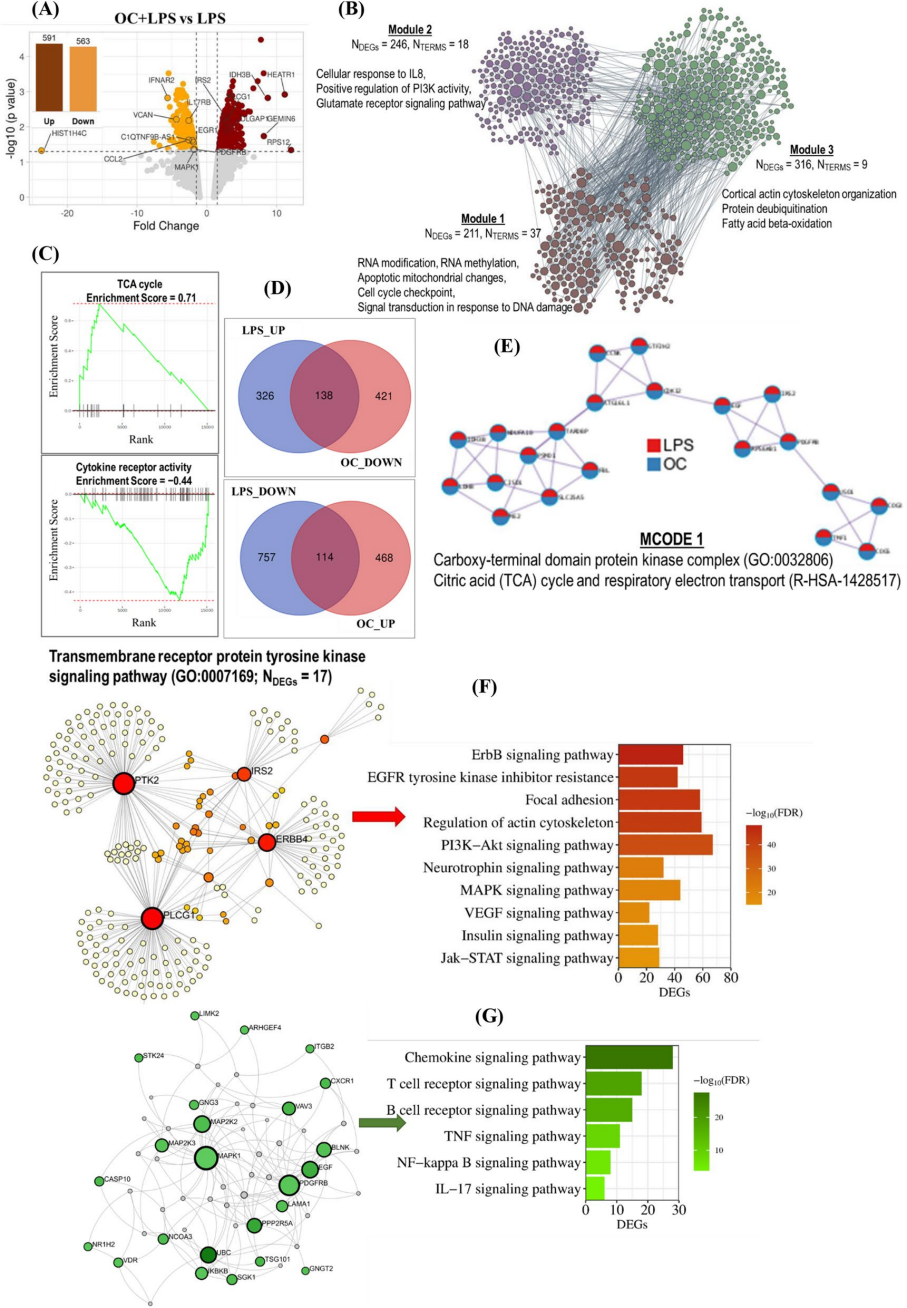
Transcriptomic response of oleacein in LPS-induced neuroinflammatory condition in SH-SY5Y cells. Cells were treated with 10 µM oleacein (OC) and 5 µg/ml LPS for 24 hours, after which RNA was isolated from both the LPS-treated group and the OC and LPS co-treated group (OC+LPS), and microarray analysis was conducted. **A** A volcano plot illustrating the differentially expressed genes (DEGs) between OC+LPS condition and LPS condition. The vertical axis (y-axis) represents the −log10 *P* value, while the horizontal axis (x-axis) displays the linear fold change. Upregulated genes are represented by brown dots, while downregulated genes are shown in orange dots. The top 20 DEGs with the largest fold changes are highlighted, and inset bar graph indicates the number of DEGs, with brown bar denoting the number of upregulated DEGs and orange bar representing the number of downregulated genes. **B** Functionally clustered modules by the DEGs based on shared k-nearest neighbors and the Louvain community-finding algorithm using the HumanBase public database. Significantly enriched Gene Ontology biological process (GOBP) terms of each module are presented, with N_TERMs_ indicating the number of enriched GOBP terms in each module and N_DEGs_ representing the number of DEGs in each module. **C** Gene Set Enrichment Analysis (GSEA) plots depict the predicted top activated KEGG pathway, TCA cycle, and top downregulated molecular function ‘Cytokine receptor activity’. **D** Venn diagrams depict the count of shared DEGs exhibiting opposite expression patterns between the LPS and OC+LPS conditions, meaning they were upregulated in one condition but downregulated in the other, and vice versa. **E** The functional network shows densely connected DEGs in both the LPS vs Control and OC+LPS vs LPS conditions created by applying the MCODE algorithm. Network nodes are displayed as pies, with red representing the LPS vs Control gene list and blue representing the OC+LPS vs LPS condition gene list. **F** Functional enrichment network of the upregulated DEGs in the OC+LPS vs LPS condition associated with the transmembrane receptor protein tyrosine kinase signaling pathway (GO:0007169). The bar graph displays the significantly enriched KEGG pathways in the network, with color coding indicating the significance (−log10 (FDR)). **G** Functional enrichment network of the downregulated DEGs in the OC+LPS vs LPS condition. The bar graph represents the significantly enriched KEGG pathways in the network, with color coding indicating the significance (−log10 (FDR))

We examined the effects of OC in an in vitro neuroinflammation model induced by LPS in SH-SY5Y cells. Compared to the LPS-induced untreated group (LPS), the group co-treated with OC and LPS (OC+LPS) exhibited differential expression in 1,154 genes (*P <* 0.05, FC > 2). Among these DEGs, 591 were upregulated and 563 were downregulated (Fig. 3A). Notably, the top upregulated DEG was ribosomal protein S12 (*RPS12*, FC = 12.07), while the top downregulated DEG was histone cluster 1, H4c (*HIST1H4C*, FC = −23.69).

We then identified functionally clustered genes based on shared k-nearest-neighbors (SKNN) and the Louvain community finding algorithm using the HumanBase public database, allowing for network-based functional interpretation of the OC-modulated DEGs under inflammatory conditions (Fig. 3B). Each module comprised tightly connected genes with a co-membership score > 0.9, and for each module, gene ontology biological process (GOBP) terms were detected for functional enrichment. Module 1, consisting of 211 functionally connected DEGs, enriched 37 GOBP terms, including RNA modification, RNA methylation, apoptotic mitochondrial changes, cell cycle checkpoint, and signal transduction in response to DNA damage. Module 2 (DEGs = 246) enriched terms related to cellular response to IL8, positive regulation of PI3K activity, and glutamate receptor signaling pathway. Module 3 (DEGs = 316) enriched terms such as cortical actin cytoskeleton organization, protein deubiquitination, and fatty acid β-oxidation.

GSEA analysis revealed that among the top enriched upregulated KEGG pathways was the citrate cycle (TCA cycle) with an ES of 0.5004 and a *P* value of 0.01. The top enriched molecular function included cytokine receptor activity (ES = −0.4305 and *P* = 0.001) (Fig. 3C).

To investigate whether OC could counteract LPS-induced gene expression, we employed Venn diagrams. We found that OC treatment downregulated the expression of 138 genes that were upregulated in the LPS-induced condition, while OC upregulated the expression of 114 genes that were downregulated in the LPS-induced condition (Fig. 3D). Furthermore, we used the MCODE algorithm to identify neighborhoods where proteins were densely connected. We observed that the commonly regulated genes in opposite directions between the LPS and OC+LPS conditions were related to the protein kinase complex and the TCA cycle (Fig. 3E). Specifically, 17 upregulated DEGs in the OC+LPS group were associated with the transmembrane receptor protein tyrosine kinase signaling pathway (GO:0007169). Functional network enrichment analysis of these 17 genes revealed significant enrichment in pathways such as the ErbB signaling pathway, EGFR tyrosine kinase inhibitor resistance, focal adhesion, PI3K-Akt signaling pathway, neurotrophin signaling pathway, MAPK signaling pathway, VEGF signaling pathway, insulin signaling pathway, and Jak-STAT signaling pathway (Fig. 3F). Conversely, functional enrichment analysis of the downregulated DEGs highlighted several inflammation-related pathways, including the chemokine signaling pathway, T cell and B cell receptor signaling pathways, TNF signaling pathway, NF-кB signaling pathway, and IL17 signaling pathway (Fig. 3G).

### Oleacein ameliorated LPS-induced depressive-like behavior in ICR mice

**Fig. 4:**
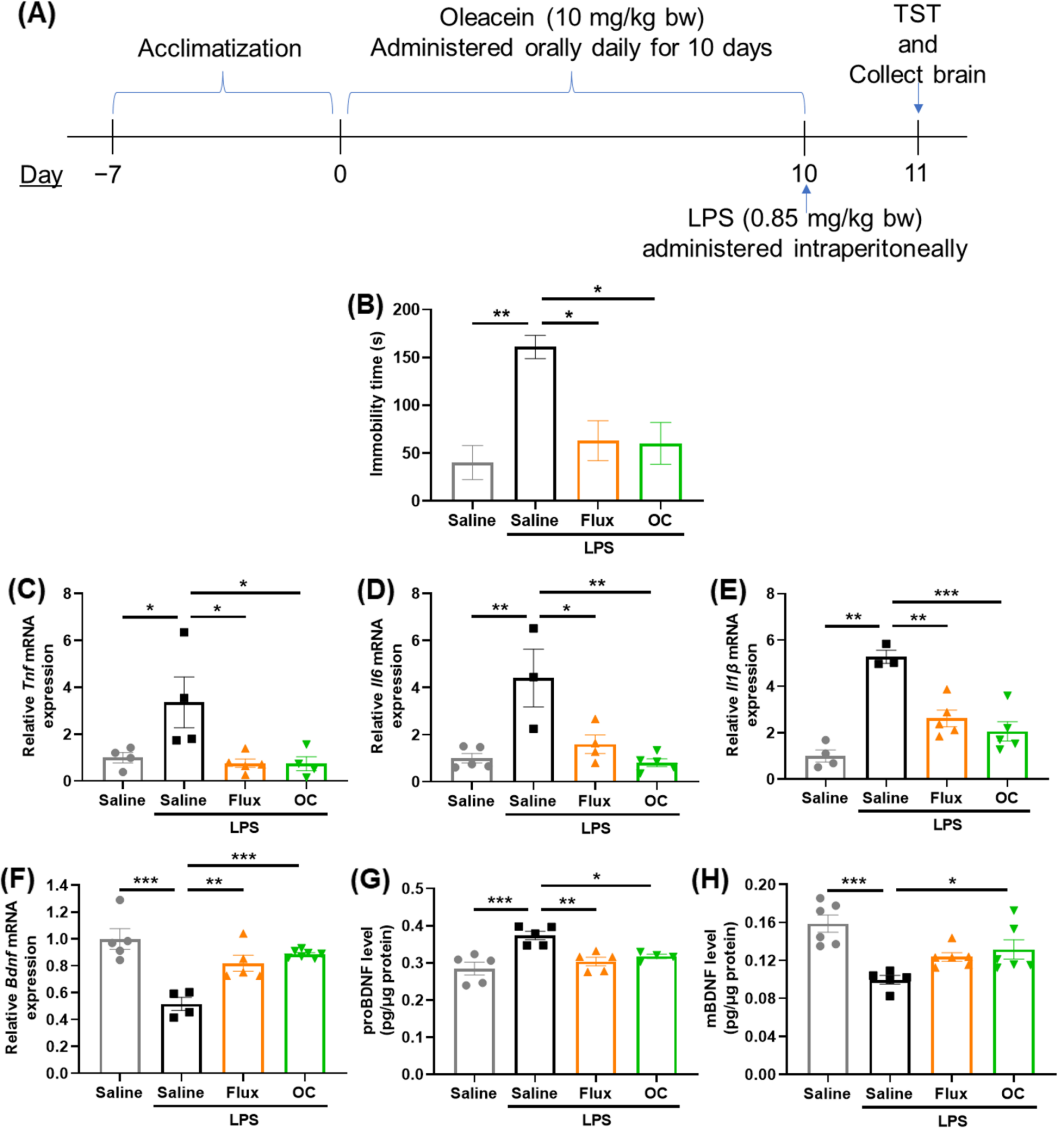
Evaluation of the effects of oleacein on depressive behavior, using the tail suspension test, in mice induced with LPS, and expression analysis of BDNF and inflammatory cytokines in the hippocampus of the brain. **A** Schedule of the behavioral test for the evaluation of the depressive state level in LPS-induced neuroinflammatory mice. **B** The immobility time in tail suspension test. The data are expressed as mean ± SEM. ^*^P < 0.05 vs LPS group. To examine the effect of OC administration on LPS-induced neuroinflammation, the expression levels of key proinflammatory cytokines **C** *Tnfα*, **D** *Il6*, and **E** *Il1β* were examined in the mouse brain hippocampus (*n* = 4~) using real-time qPCR. To examine the effect of OC administration on neurogenesis, **F** *Bdnf* mRNA expression by real-time qPCR, and **G** proBDNF and **H** mBDNF protein expressions by ELISA were examined (*n* = 4~). *P < 0.05, **P < 0.01, ***P < 0.001 compared to control group, by one-way ANOVA followed by Tukey’s post hoc test

To assess the efficacy of OC in alleviating depressive-like symptoms triggered by inflammation, we employed an LPS-induced depression-like model in mice. ICR mice were subjected to oral OC administration for 10 consecutive days. Subsequently, a single intraperitoneal injection of LPS was administered, and the mice's depressive behavior was assessed by measuring immobility time in the TST (Fig. 4A). We used Fluoxetine, a well-known antidepressant drug belonging to the selective serotonin reuptake inhibitors group, as a positive control to compare the effects of OC on depressive behavior.

LPS-injected ICR mice exhibited a significant increase in immobility time (160.96 + 24.31s; *P* = 0.003) compared to the saline-administered mice in the control group (40.13 + 39.68s). However, oral administration of OC for 10 days prior to the LPS injection could significantly attenuate the LPS-induced increase in immobility time (60.21 + 48.77s; *P* = 0.013), which was even lower than that of the positive control Flux group (62.96 + 46.57s; *P* = 0.016) (Fig. 4B).

### Oleacein reduced the mRNA expression of pro-inflammatory cytokines in the *hippocampus* of mice induced by LPS

We examined the effects of OC administration on proinflammatory cytokines in the hippocampus of LPS-induced mice utilizing RT-qPCR analysis. LPS administration led to a significant increase (3.36 ± 2.16; *P* = 0.047) in Tnf mRNA expression compared to the control group (1.00 ± 0.45) (Fig. 4C). Nevertheless, this increase was notably suppressed by OC administration (0.74 ± 0.60; *P* = 0.026), which was comparable to the positive control Flux group (0.76 + 0.41; *P* = 0.02). Similarly, LPS administration significantly increased *Il6* expression (4.40 ± 2.13; *P* = 0.002) compared to the control group (1.00 ± 0.45) (Fig. 4D), which was significantly mitigated by OC administration (0.81 ± 0.37; *P* = 0.001). Flux administration reduced *Il6* expression as well (1.60 ± 0.79; *P* = 0.011). Finally, regarding Il1β mRNA, LPS administration resulted in a significant increase (5.27 ± 0.49; *P <* 0.001) compared to the control group (1.00 ± 0.52) (Fig. 4E). Yet, OC administration significantly suppressed this increase (2.06 ± 0.93; *P <* 0.001), which was comparable to the positive control Flux group (2.62 ± 0.80; *P =* 0.002). These results suggest that OC could mitigate LPS-induced neuroinflammation in mice brain.

### Oleacein increased BDNF expression in mice *hippocampus*

Next, we examined the effects of OC administration on *Bdnf* expression in the hippocampus of LPS-induced mice utilizing RT-qPCR analysis (Fig. 4F). The findings revealed that LPS administration significantly reduced (0.52 ± 0.10; *P <* 0.001) the expression of *Bdnf* compared to the control group (1.00 ± 0.17). However, similar to Flux group (0.82 ± 0.13; *P =* 0.005), OC treatment significantly reversed this decrease (0.89 ± 0.03; *P <* 0.001) (Fig. 4G).

Recently, there has been growing interest in the role of proBDNF, a precursor of mature BDNF (mBDNF), in the central nervous system. ProBDNF specifically binds to the p75 neurotrophin receptor (p75NTR), leading to neuronal apoptosis and enhanced long-term depression (LTD) of neurotransmission. Notably, proBDNF exhibits biological responses that are opposite to those of mBDNF [31]. Therefore, we measured both proBDNF and mBDNF levels in the mouse hippocampus using commercially available ELISA kits (Fig. 4G, H).

The results showed that LPS injection significantly increased proBDNF levels (0.37 ± 0.03 pg/μg protein; *P <* 0.001) compared to the control group (0.28 ± 0.04 pg/μg protein). However, OC administration significantly attenuated this increase (0.32 ± 0.02 pg/μg protein; *P* = 0.038), which was comparable to Flux group (0.30 ± 0.03; *P* = 0.006). On the other hand, mBDNF levels were significantly reduced (0.10 ± 0.01 pg/μg protein; *P <* 0.001) by LPS injection compared to the control group (0.16 ± 0.02 pg/μg protein). However, OC administration significantly reversed this decrease (0.13 ± 0.03 pg/μg protein; *P* = 0.048), comparable to Flux group (0.12 ± 0.01; *P* = 0.18).

### Transcriptomics analysis showed oleacein mitigated neuroinflammation in mice brain *hippocampus*

**Fig. 5:**
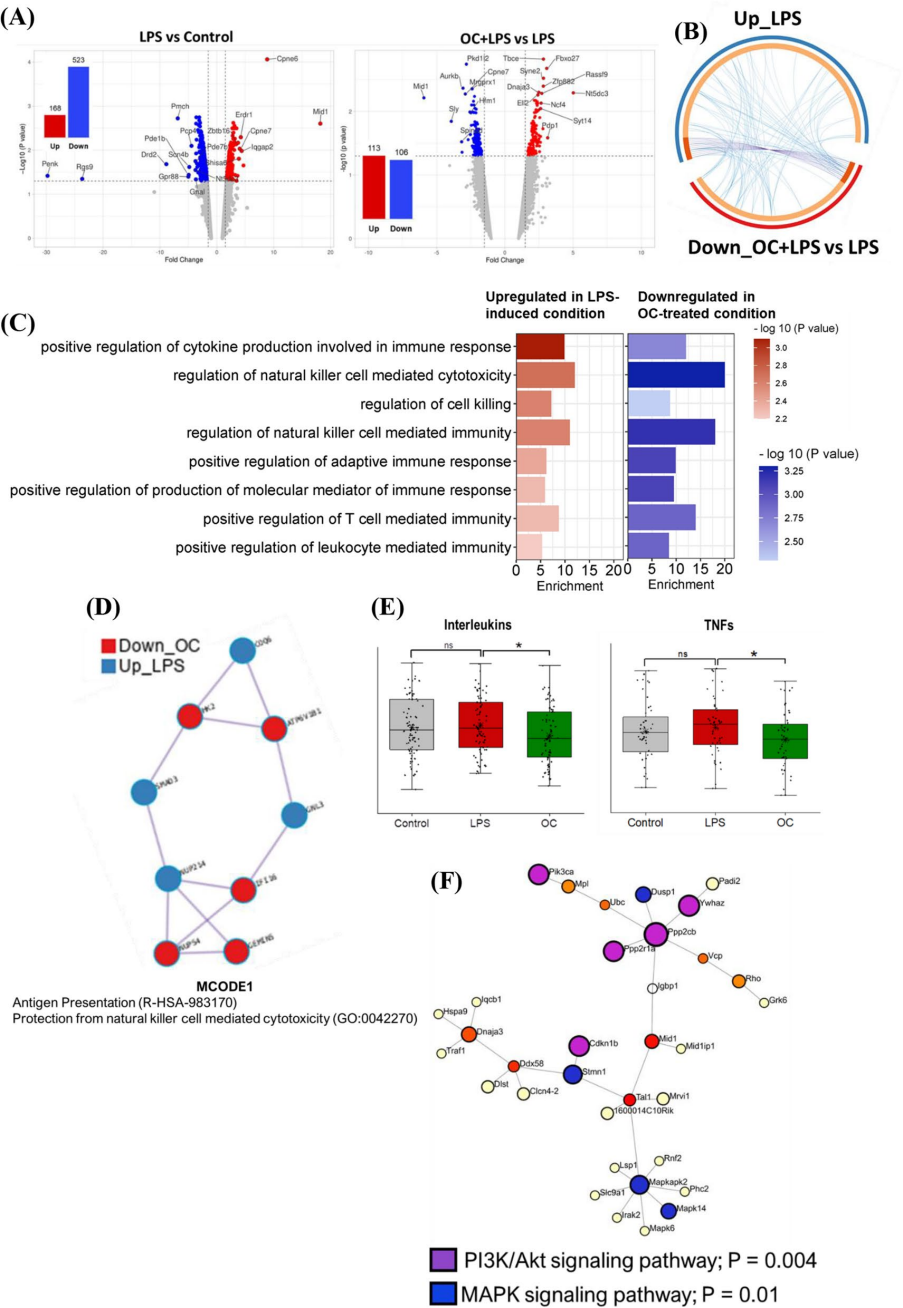
Transcriptome-wide effects of oleacein in mice hippocampus of LPS-induced depression model mice. **A** Volcano plots illustrating the differentially expressed genes (DEGs) between LPS vs control conditions and OC+LPS vs LPS conditions. The vertical axis (y-axis) represents the −log10 *P* value, while the horizontal axis (x-axis) displays the linear fold change. Upregulated genes are depicted in red dots, whereas downregulated genes are shown in blue dots. The top 20 DEGs with the largest fold changes are highlighted, along with inset bar graphs indicating the number of DEGs. The red bars denote the count of upregulated DEGs, while the blue bars represent the count of downregulated genes. **B** The Circos plot shows how the upregulated genes between LPS vs Control condition overlap with the downregulated genes between OC+LPS vs LPS condition. The outer part of the plot displays two arcs: a blue arc representing the list of upregulated DEGs from the LPS vs Control comparison, and a red arc representing the downregulated DEG list from the OC+LPS vs LPS comparison. Inside, dark orange indicates DEGs shared by both conditions, while light orange represents DEGs unique to each condition. Purple lines link the same genes shared by both conditions, and blue lines connect DEGs that, though different, fall under the same significantly enriched GO term. **C** The bar graphs illustrate the Gene Ontology Biological Process (GOBP) terms that exhibited significant overrepresentation by the upregulated DEGs in the LPS vs Control condition and by the downregulated DEGs in the OC+LPS vs LPS comparison. The color code indicates the significance (−log 10 (*P* value)). **D** A functional enrichment network was constructed using the MCODE algorithm. Network nodes are depicted as pies, where red represents the OC+LPS vs LPS downregulated gene list, and blue represents the LPS vs Control upregulated gene list. (E) Box plots are presented to visualize the signal intensity of interleukins and TNFs across the control, LPS, and OC (OC+LPS) conditions. *P < 0.05, Two-way unpaired t-test. **F** Functional enrichment analysis (KEGG pathway) encompassing all significant genes (*P <* 0.05). Purple nodes refer to the key genes involved in PI3K/Akt pathway, while blue nodes refer to the key genes associated with MAPK pathway (OC+LPS vs LPS condition)

Next, we investigated the transcriptomic changes in the mouse hippocampus induced by OC in response to LPS-induced neuroinflammation. A total of 22,203 probe IDs were identified in the Clariom S Assay Mouse Array. Comparing the LPS and control (saline-administered) groups revealed differential expression in 691 genes (*P* value < 0.05, FC > 2), with 168 upregulated and 523 downregulated genes. Conversely, between the OC+LPS and LPS groups, 219 genes showed differential expression, with 113 significantly upregulated and 106 downregulated (Fig. 5A).

The Circos plot offers a comprehensive visualization of gene and functional overlaps between different conditions: LPS-induced vs control and OC-treated LPS-induced vs LPS conditions (Fig. 5B). The outer part of the plot displays two arcs: a blue arc representing the list of upregulated DEGs from the LPS vs control comparison and a red arc representing the downregulated DEG list from the OC+LPS vs LPS comparison. The plot illustrates the DEGs shared between both conditions in dark orange, indicating common genes with expression in opposite directions. Additionally, light orange signifies DEGs unique to each condition. Purple lines link the common DEGs shared by both conditions, while blue lines connect DEGs falling under the same significantly enriched GO term. There were 11 genes that directly overlap between the conditions, i.e., upregulated by LPS but downregulated by OC treatment. However, functional overlap is also evident, as indicated by the light blue lines.

Further analysis of the overrepresented GOBP terms in both LPS vs control and OC+LPS vs LPS conditions revealed significant inflammation-related biological events in the upregulated gene list of the LPS vs control condition, while the exact BP terms were overrepresented in the downregulated gene list of the OC+LPS vs LPS condition. These GOBP terms included positive regulation of cytokine production in immune response, positive regulation of T cell-mediated and leukocyte-mediated immunity, etc. (Fig. 5C).

Applying the MCODE algorithm to the functional network, we observed that antigen presentation and natural killer cell-mediated cytotoxicity were upregulated in the LPS condition but downregulated in the OC-treated condition (Fig. 5D). Further examination of the signal intensity of interleukins and TNFs probe IDs in all three groups (control, LPS, and OC+LPS) revealed an increase in signal intensity in the LPS condition compared to the control condition, although not statistically significant. However, the signal intensity of interleukins and TNFs was significantly decreased in the OC-treated condition compared to the LPS group (Fig. 5E). To explore further potential mechanism, we included all significant genes with a *P*-value < 0.05 for KEGG pathway functional enrichment analysis, which unveiled the involvement of the PI3K/Akt and MAPK pathways, with the former showing greater significance (Fig. 5F).

## Discussion

In the present study, we conducted a series of investigations both in vitro and in vivo and explored the potential of the rare olive compound OC as a TrkB agonist. Our findings suggest its efficacy in mitigating neuroinflammation in a depression-like mouse model by potentially modulating the BDNF-TrkB axis.

In recent years, BDNF has garnered attention for its crucial role in regulating the neuroimmune axis in mental health disorders. The neurotrophic activity of BDNF/TrkB is believed to modulate the intricate interaction between neuroinflammation and neurogenesis in the pathogenesis of depression-like disorders [8, 32–34], making it a promising therapeutic target for such conditions. Upon binding to BDNF, TrkB isoforms become activated and initiate various downstream signal transduction pathways at the intracellular level. These pathways include PI3/Akt/mTOR, Ras-Raf-MEK-ERK, and PLCγ1/PKC, which play crucial roles in promoting neuronal cell proliferation and differentiation [35, 36]. Conversely, the functions related to cell survival and apoptosis within the BDNF/TrkB transduction pathways are predominantly governed by downstream mechanisms such as NFκB pathway [37, 38] and JAK/STAT pathway [39–41]. In the present study, we, therefore, focused on examining the effects of oleacein on the BDNF/TrkB axis and its role in modulating inflammatory cytokine levels both in vitro and in vivo.

In our investigation, we utilized the SH-SY5Y cell line as a first-line screening tool to explore the potential bioactivities of oleacein in vitro. SH-SY5Y cells are widely recognized in neuroscience, and LPS-treated cells serve as a classic in vitro model for neuroinflammation [42, 43]. The main advantage of using SH-SY5Y cells is that they are human-derived, allowing us to avoid interspecies differences and achieve results that are more relevant to human physiology, thereby making the findings more applicable to human biology and potential therapeutic applications. We observed a dose-dependent increase in *BDNF* expression in human neuroblastoma SHSY5Y cells upon treatment with OC (Fig. 1A). However, when inhibitors of TrkB, PI3k, and MEK were introduced, this increase was notably absent (Fig. 1B), suggesting the involvement of OC in BDNF/TrkB signaling and its downstream pathways.

Transcriptomic analyses in both normal and LPS-induced conditions in SH-SY5Y cells revealed how OC affects BDNF/TrkB neurotrophic activities (Figs. 2, 3). Under normal conditions, OC primarily exhibited functions associated with the cell cycle (Fig. 2B, D, E, and H), while inhibiting a subset of inflammatory functions (Fig. 2C). Furthermore, OC treatment upregulated several BDNF-coexpressed genes in normal conditions (Fig. 2F), particularly those involved in neuron differentiation and axonogenesis (Fig. 2G). Conversely, in LPS-induced condition in SH-SY5Y cells, OC was found to modulate various inflammatory pathways in addition to cell cycle-related functions (Fig. 3B, C, and E). Notably, OC treatment counteracted LPS-induced biological events (Fig. 3E) and gene expression alterations (Fig. 3D). Specifically, OC upregulated numerous transmembrane receptor protein tyrosine kinases, significantly influencing downstream PI3K/Akt, MAPK, and Jak/STAT signaling pathways (Fig. 3F). Conversely, OC treatment downregulated NFκB, TNF, and IL17 pathways (Fig. 3G).

We further validated these findings in vivo. Advanced Akaluc BLI revealed that a single oral administration of OC significantly enhanced BDNF expression in the mouse brain (Fig. 1C, D). Next, we explored the antidepressant-like effects of OC in a mouse model of LPS-induced depression-like symptoms, using the tail suspension test (TST), a commonly employed behavioral test to assess pharmacological antidepressant activity in animals [44]. We observed that oral administration of OC for seven days effectively reduced the immobility time in the TST following LPS administration (Fig. 4B). Additionally, OC treatment significantly downregulated the expression of major proinflammatory cytokines, including *Tnf*, *Il6*, and *Il1β*, in the mice hippocampus (Fig. 4C-E). Most importantly, OC administration led to a significant increase in *Bdnf* expression (Fig. 4F), and subsequent protein expression analysis revealed upregulation of mBDNF and downregulation of proBDNF levels (Fig. 4G, H). BDNF has been reported to exhibit direct modulatory effects on LPS-induced inflammation in both microglia and neuronal cultures [9]. Furthermore, in vivo studies have demonstrated that the administration of pro-inflammatory cytokines or lipopolysaccharide (LPS) significantly reduces mature BDNF levels in brain regions such as the hippocampus and cerebral cortex [8]. Additionally, numerous studies suggest that BDNF may play a potential role in depression by influencing neurogenesis and synaptic plasticity [45]. Our study findings suggest that OC may possess potential antidepressant-like functions by promoting neurogenesis and suppressing neuroinflammation. However, depression is a multifaceted condition involving various pathologies. Further research is necessary to confirm the effects of OC on depression or anxiety, encompassing a comprehensive evaluation of the biological events associated with depression, including neurotransmitter levels.

Furthermore, DNA microarray analysis predicted that the primary response to OC treatment was the suppression of LPS-induced inflammation in the hippocampus of mice (Fig. 5C-E). Further network enrichment analysis, encompassing all significant genes, indicated significant enrichment of both the PI3K/Akt and MAPK pathways, with the former exhibiting greater significance (Fig. 5F). Future research aimed at these pathways would validate OC's target pathways in alleviating depression-like behavior. It is worth mentioning that while chronic stress-induced depression-like behavior serves as a conventional mouse model for studying depression, LPS administration has been reported to be more effective in inducing depressive behavior in TST and increasing inflammatory cytokine levels compared to unpredictable chronic mild stress (UCMS) [46], underscoring the importance of considering LPS-induced model when evaluating the effects of target compounds on the neuroimmune axis of depression.

Additionally, we explored the affinity of OC for the TrkB receptor. OC exhibited superior affinity for the TrkB receptor compared to the positive control 7,8-DHF, indicating robust pharmacological activity at low KD values (Fig. 1E, F). The TrkB agonist activity of 7,8-DHF has been attributed to its catechol group [47]. OC possesses a distinctive chemical structure characterized by a catechol group and two aldehyde groups, setting it apart from other olive leaf compounds. This unique chemical configuration likely accounts for the potent binding activity of OC to the TrkB receptor. Notably, even upon exposure to regeneration fluid, OC remained bound to the TrkB receptor without dissociation. This suggests that OC maintains stable binding to the TrkB receptor, potentially leading to sustained activation of its downstream signaling pathways.

**Fig. 6:**
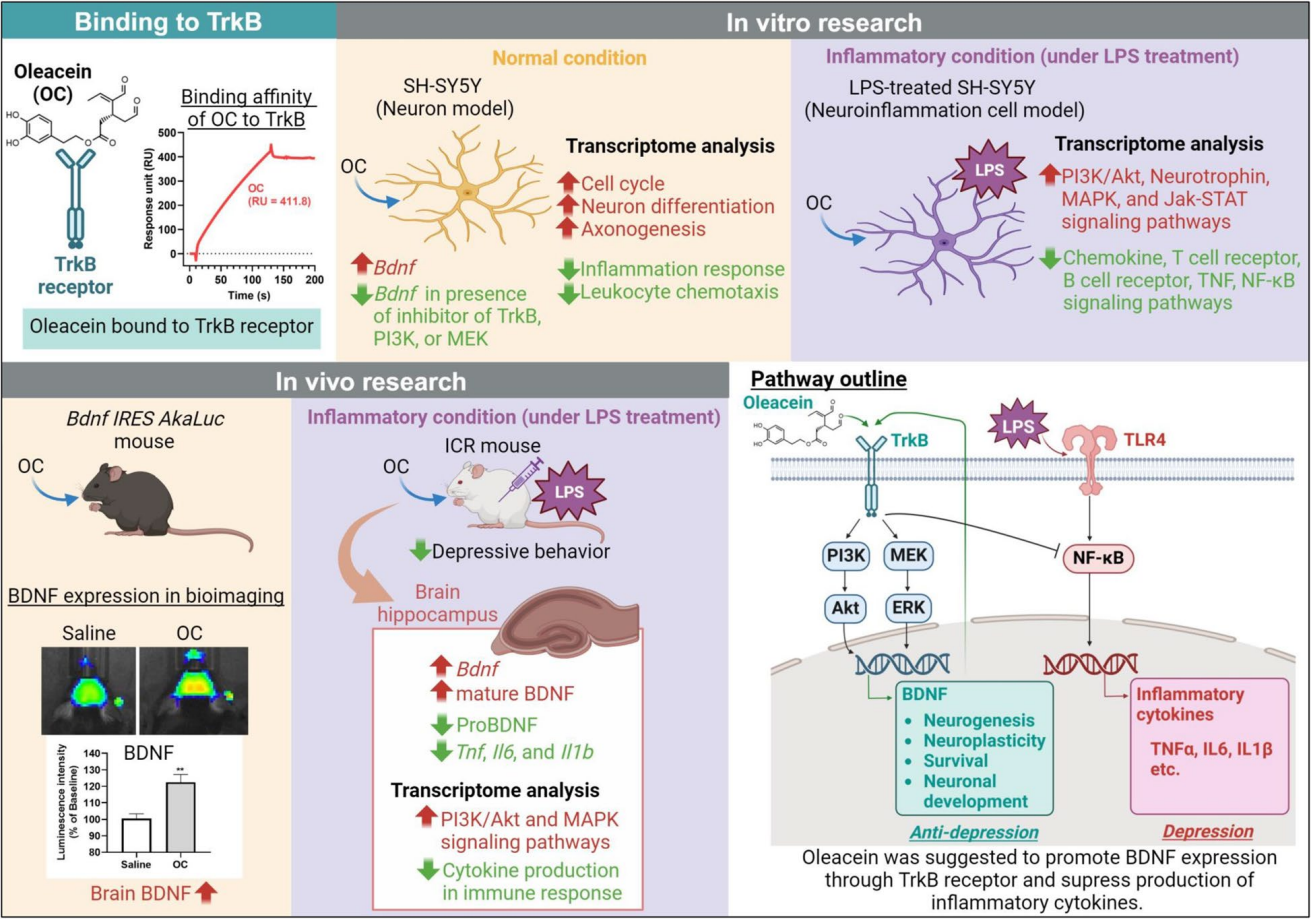
Summary of the evidence on potential neurogenesis-promoting and neuroinflammation-mitigating effects of oleacein

Due to its rarity, OC has not received much research attention despite its promising bioactivities. However, a recent breakthrough by a research group has managed to chemically synthesize OC from commercially available OP with a high yield [18], sparking renewed interest in its potential. Our study marks the first exploration of OC's function as a natural TrkB agonist, demonstrating its ability to enhance neurogenesis and alleviate neuroinflammation in both in vitro and in vivo models (Fig. 6). Collectively, our findings propose that OC holds promise for innovative therapeutic approaches targeting depression-like mental disorders.

## Data Availability

Microarray data are publicly accessible in the  Gene Expression Omnibus (GEO) repository. The datasets can be retrieved under the accession numbers GSE263605 (in vitro) and GSE263606 (in vivo).
